# A micro-fluidic study of whole blood behaviour on PMMA topographical nanostructures

**DOI:** 10.1186/1477-3155-6-3

**Published:** 2008-02-19

**Authors:** Caterina Minelli, Akemi Kikuta, Nataliya Tsud, Michael D Ball, Akiko Yamamoto

**Affiliations:** 1 International Centre for Young Scientists, National Institute for Materials Science, 1-1 Namiki, Tsukuba, Ibaraki 305-0044, Japan; 2 Department of Materials, Imperial College London, Exhibition road, SW7 2AZ, London, UK; 3 Biomaterial Centre, National Institute for Materials Science, 1-1 Namiki, Tsukuba, Ibaraki 305-0044, Japan; 4 Nanomaterials Laboratory, National Institute for Materials Science, 1-1 Namiki, Tsukuba, Ibaraki 305-0044, Japan

## Abstract

**Background:**

Polymers are attractive materials for both biomedical engineering and cardiovascular applications. Although nano-topography has been found to influence cell behaviour, no established method exists to understand and evaluate the effects of nano-topography on polymer-blood interaction.

**Results:**

We optimized a micro-fluidic set-up to study the interaction of whole blood with nano-structured polymer surfaces under flow conditions. Micro-fluidic chips were coated with polymethylmethacrylate films and structured by polymer demixing. Surface feature size varied from 40 nm to 400 nm and feature height from 5 nm to 50 nm. Whole blood flow rate through the micro-fluidic channels, platelet adhesion and von Willebrand factor and fibrinogen adsorption onto the structured polymer films were investigated. Whole blood flow rate through the micro-fluidic channels was found to decrease with increasing average surface feature size. Adhesion and spreading of platelets from whole blood and von Willebrand factor adsorption from platelet poor plasma were enhanced on the structured surfaces with larger feature, while fibrinogen adsorption followed the opposite trend.

**Conclusion:**

We investigated whole blood behaviour and plasma protein adsorption on nano-structured polymer materials under flow conditions using a micro-fluidic set-up. We speculate that surface nano-topography of polymer films influences primarily plasma protein adsorption, which results in the control of platelet adhesion and thrombus formation.

## Background

Blood compatibility of materials is one of the major issues of medical engineering. Devices for cardiovascular applications are widely used but still do not exhibit optimal performances and must be often combined with anticoagulation drugs, with important implications for patient health and therapy costs [1]. The techniques available to evaluate the blood compatibility of materials to date are still limited, in spite of the heavy demand for methods allowing the quantitative and accurate characterization of the polymers used in the construction of cardiovascular devices [2].

The difficulties encountered in characterising the interaction of blood with materials are due principally to the complexity of the phenomena involved. The adsorption and subsequent conformational changes of proteins from the blood are the first events that take place when blood contacts an artificial material. Then, platelets adhere over the adsorbed protein layer, react with it and thrombus formation is eventually initiated [1,3]. Many surface modification techniques [4,5] such as chemical treatment [6,7] or specific molecular immobilization [8-11] have been explored to control polymer interaction with blood cells and proteins.

The interaction of blood with the materials' surface involves mechanisms that occur at different length scales. Interestingly, natural tissue surfaces such as blood vessels exhibit features in the nanometer range and nano-topography has been found to influence cell behaviour, including morphology [12], adhesion [13] and motility [14] for a number of cells [12,13,15-20], in static and flow [21] conditions. It is still unclear though to what extent surface topography influences blood behaviour and, therefore, materials' blood compatibility, and what the mechanisms involved are. To our knowledge no systematic studies were conducted to understand the effects of nano-topography on blood-polymer interaction. In this work we describe a micro-fluidic set-up for investigating the interaction of blood with polymer nano-structured surfaces under flow conditions and we provide some basic data on the platelet adhesion and plasma protein adsorption on nano-structured polymethylmethacrylate (PMMA) surfaces.

**Figure 1:**
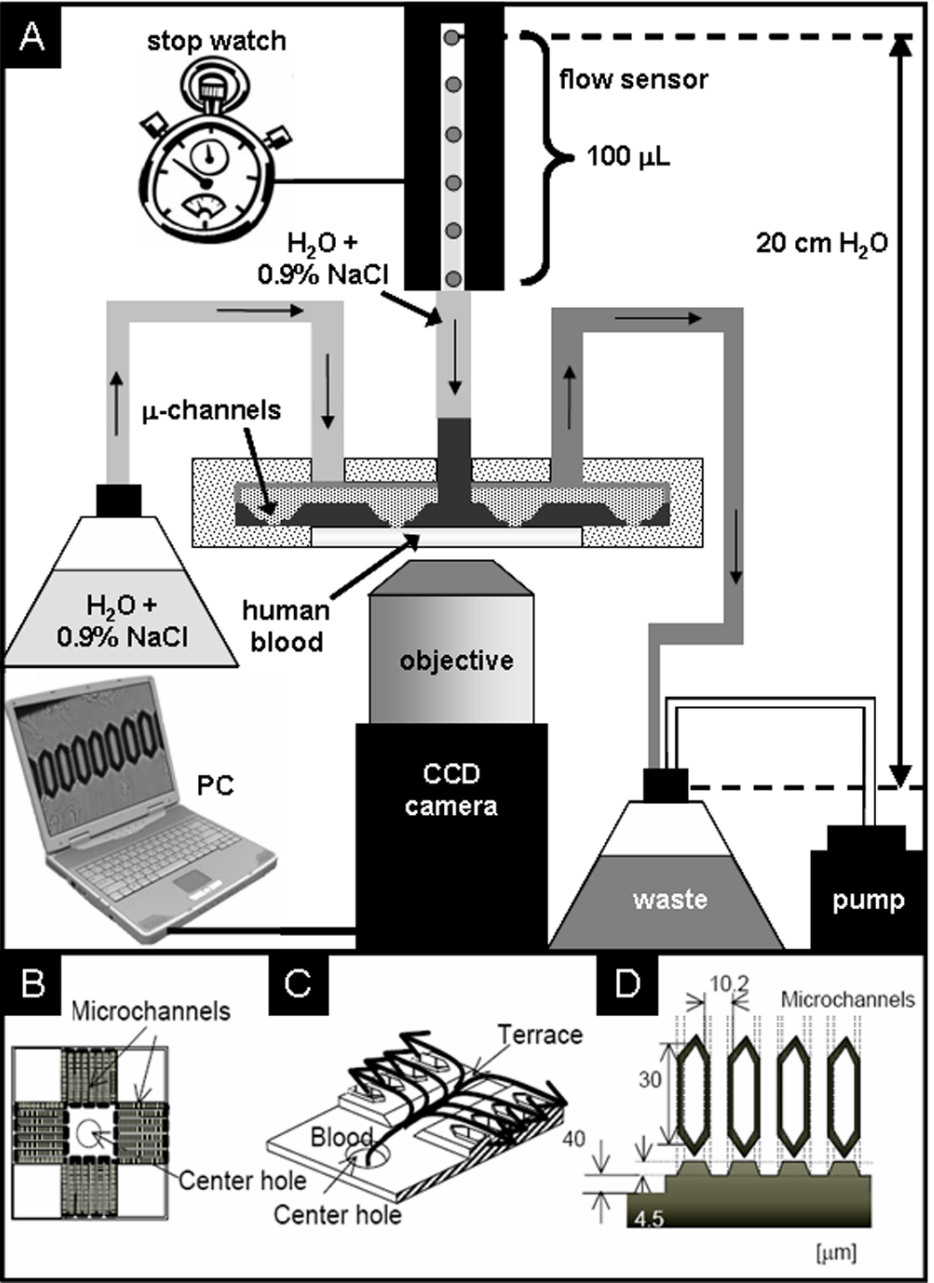
**The Micro-fluidic experiment set up**. (A) Schematic of the MC-FUN set up (not to scale). (B) Top view of a micro-fluidic chip (15 mm × 15 mm). (C) Particulars of the micro-channels. (D) Geometrical parameters of the micro-fluidic chip.

The study is performed by using a Micro-Channel Array Flow Analyzer (MC-FAN, Figure 1A), which was previously utilized to characterize the interaction of whole blood and plasma proteins with metal surfaces providing interesting insights into the importance of surface energy on blood coagulation mechanism [22]. Our intention is to demonstrate that the MC-FAN is a viable *in vitro *set-up for the study of the interaction of blood with a large class of polymers and surfaces and thus for a first-stage selection of potential blood compatible materials, avoiding the costs, the long times and the sacrifice of animals required by *in vivo *experiments.

This study was approved by the Ethics Committee of NIMS.

## Results

### Topographical and Chemical Characterization of the Surfaces

**Figure 2:**
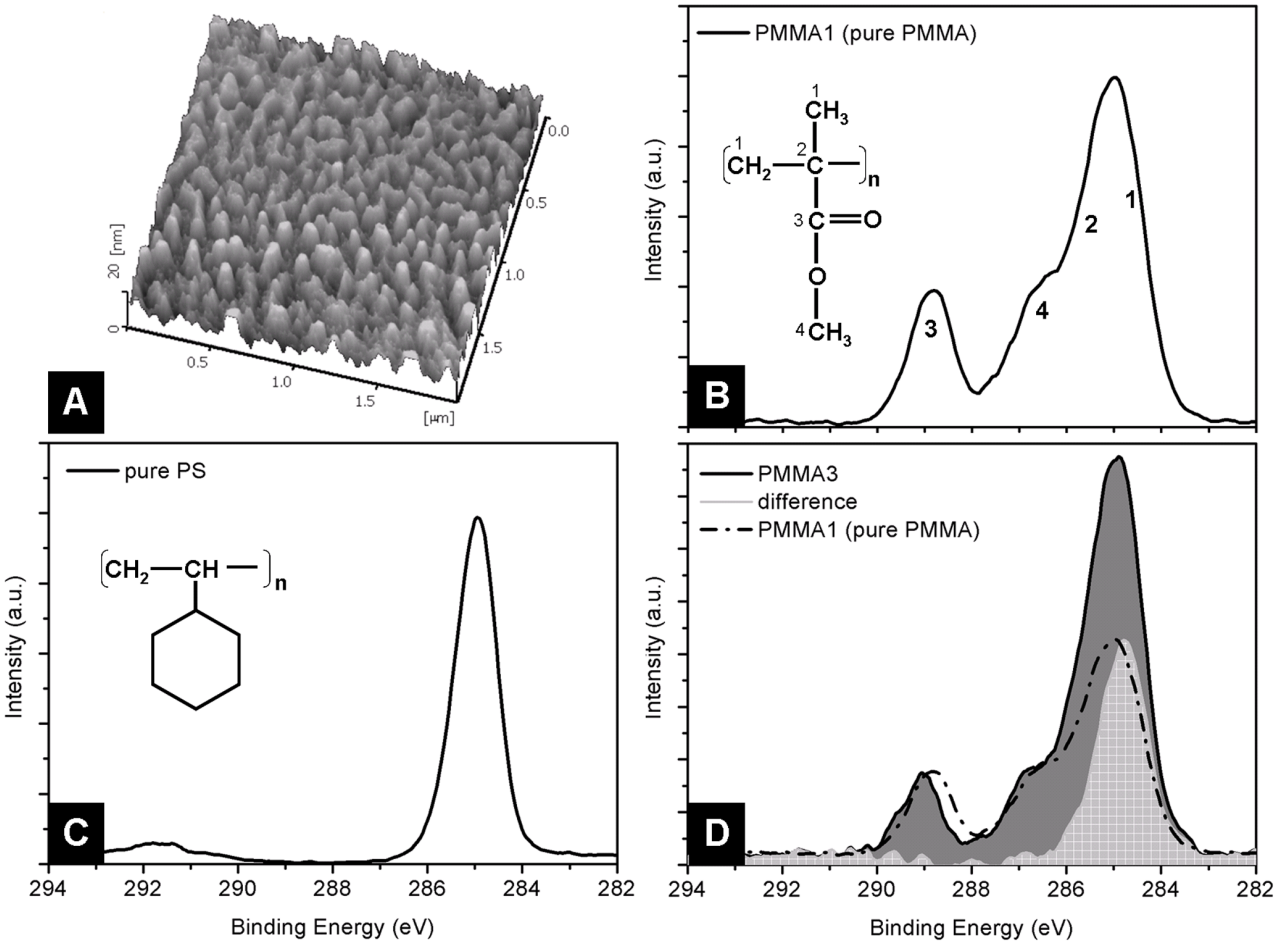
**Characterization of the structured PMMA films**. (A) AFM topographical image of PMMA3 surface, structured using the polymer demixing technique. (B) XPS C_1s _spectrum of a surface of pure PMMA. The spectrum is the result of the convolution of four peaks, indicated in numbers on the PMMA molecules. (C) XPS C_1s _spectrum of a pure PS surface. (D) XPS C_1s _spectrum of a surface similar to PMMA3 (black line). For comparison the spectrum relative to pure PMMA is shown (dashed line), together with the difference between the two spectra (in gray), computed overlapping the ester peaks at 289.1 eV of the two spectra, that contain the contribution of the solely PMMA.

Polymer demixing is a well known technique to study the response of cells to nano-topography [20] and was used in this work to create a set of nano-structured polymer surfaces having typical feature sizes between 40 nm and 400 nm. Briefly, polymer films are spin coated from a blend of polystyrene (PS) and PMMA. Due to their immiscibility, the two polymers form separate phases during solvent evaporation. The PS phase is subsequently removed by selective solvent treatment, leaving a structured PMMA film. The geometry of the PMMA surface structures is controlled varying the experimental parameters such as polymer concentration in solution and spin velocity. This technique is fast, inexpensive and particularly suitable for the fabrication of nano-structured films onto surfaces having complex geometries [23] such as the micro-fluidic chips used in this work (Fig. 1B, C and 1D). A typical Atomic Force Microscopy (AFM) topographical image of a PMMA film surface structured by polymer demixing is shown in Figure 2A. The average feature size and height were estimated from both AFM topographical and section images of the film surfaces, and their values represent the distance between the centre of a feature and the centre of a valley between two features. The film thickness was measured from AFM section profiles after having scratched the film with Teflon tweezers. The AFM measurements were performed on different areas of the same film and on similar films; the average measured values are shown in Table 1. Film thickness varied from 10 nm to 50 nm.

**Table 1:** PMMA film coating parameters and AFM characterization.

PMMA Sample	[Polymer] in toluene (%)	Feature size (nm)	Feature height (nm)
1	1.0	-	-
2	0.1	40 ± 15	3 ± 1
3	1.0	80 ± 20	13 ± 7
4	3.0	400 ± 200	50 ± 30

**Figure 3:**
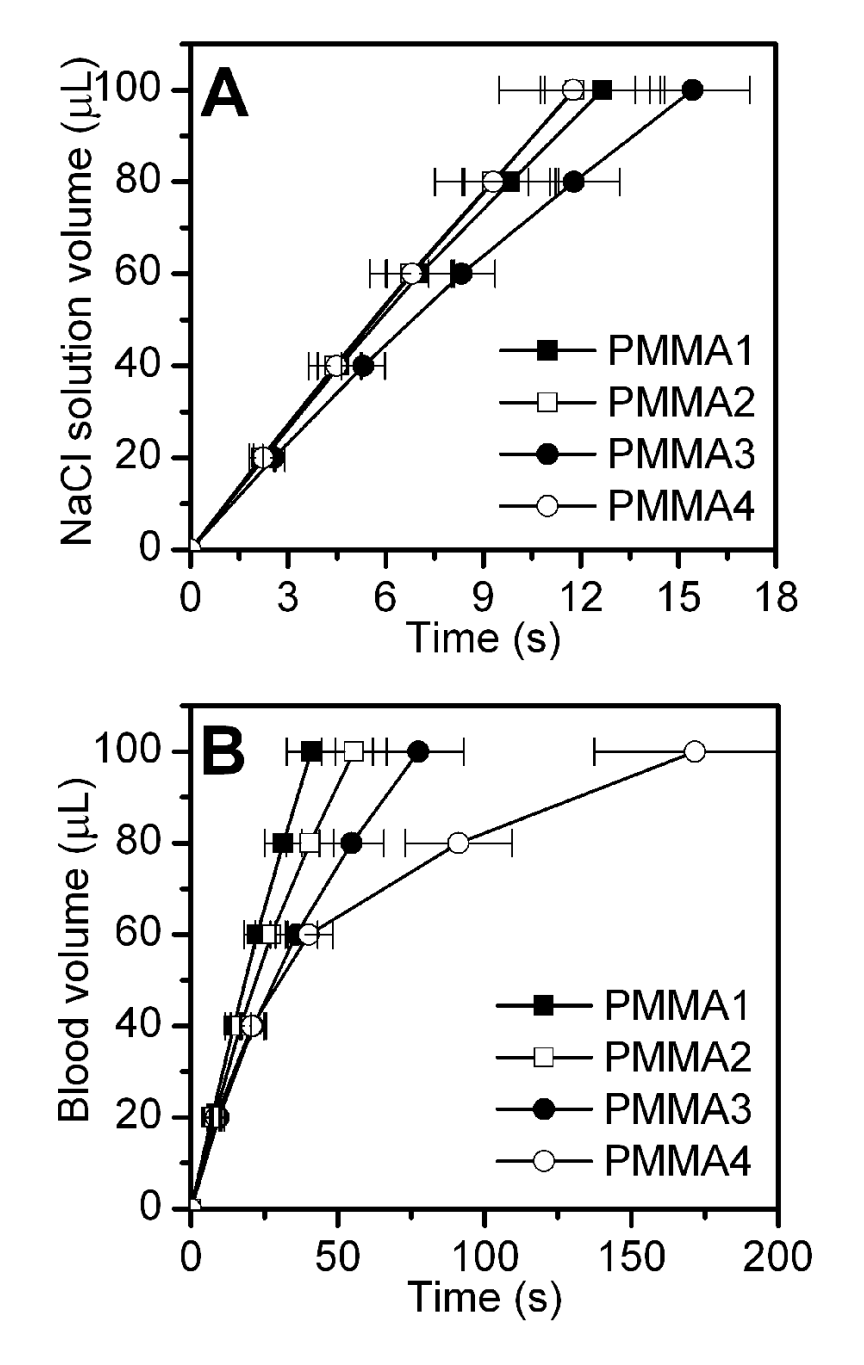
**Whole blood flow rate in micro-fluidic experiments**. Volume flow rate of (A) NaCl solution and (B) whole human blood measured with the MC-FAN on micro-fluidic chips coated with PMMA films presenting different surface topographies, as reported in Table 1.

X-ray Photoelectron Spectroscopy (XPS) measurements on samples prepared as PMMA1 (pure PMMA), PMMA2, PMMA3, PMMA4 and on pure PS films were performed to study the chemical composition of the films at the polymer/blood interface. Figure 2B shows the typical XPS C_1s _spectrum of a flat PMMA film (PMMA1). The C_1s _spectrum of pure PMMA is the result of the convolution of four peaks: the hydrocarbon (C-C/C-H) at a binding energy of 285.0 eV, the β-shifted carbon (due to their juxtaposition to O-C=O groups) at 285.7 eV, the methoxy group carbon at 286.8 eV and the carbon in the ester group at 289.1 eV [24]. The C_1s _spectrum of pure PS (Fig. 2C) includes a main hydrocarbon peak at binding energy of 285.0 eV. Figure 3D shows the typical C_1s _spectra of a structured PMMA surface. For comparison, the spectrum relative to pure PMMA is also shown (dashed line). As the ester peak at 289.1 eV contains contribution solely from PMMA, the spectrum of pure PMMA was normalized to the spectrum of each structured PMMA film to overlap the peaks relative to the ester group. From this the difference between the spectra was computed. In the case of the PMMA3 surface, this difference is shown in grey in Figure 2D and is attributed to the presence of PS hydrocarbon groups at or close to the surface. Computational analysis of the XPS spectra allowed the calculation of the composition of a 10 nm-thick layer of the polymer film at the polymer/air interface. PMMA was found to constitute (84 ± 16)%, (77 ± 15)% and (71 ± 14)% of respectively sample PMMA2, PMMA3 and PMMA4 films.

### Experiment performed with whole blood

Figure 3A shows the volume flow rate of a NaCl solution (0.9% NaCl in MilliQ water) through micro-channels coated with structured PMMA films. Error bars represent the maximum and minimum values recorded from repeated experiments. The volume which flows through the channels varies linearly with time and does not depend on the geometry of the polymer film. Figure 3B shows the same measurements using human whole blood: in this case the velocity of the blood flow volume decreases with increasing surface structure size. During the blood flow measurements, platelets were seen adhering onto the material surfaces, aggregating and eventually obstructing the micro-channels. This obstruction slowed down the blood volume rate through the micro-array chip. This slow down was used as an indicator of the quality of blood interaction with the material. The shear stress that the blood components experience is controlled by varying the pressure under which the blood flows. For example, 100 μL of human whole blood under a pressure of 2.0 kPa were measured to take (41 ± 8) s to pass through the micro-channel array of the chip coated with a flat PMMA layer, that signifies a shear rate of (3700 ± 700) s^-1^.

**Figure 4:**
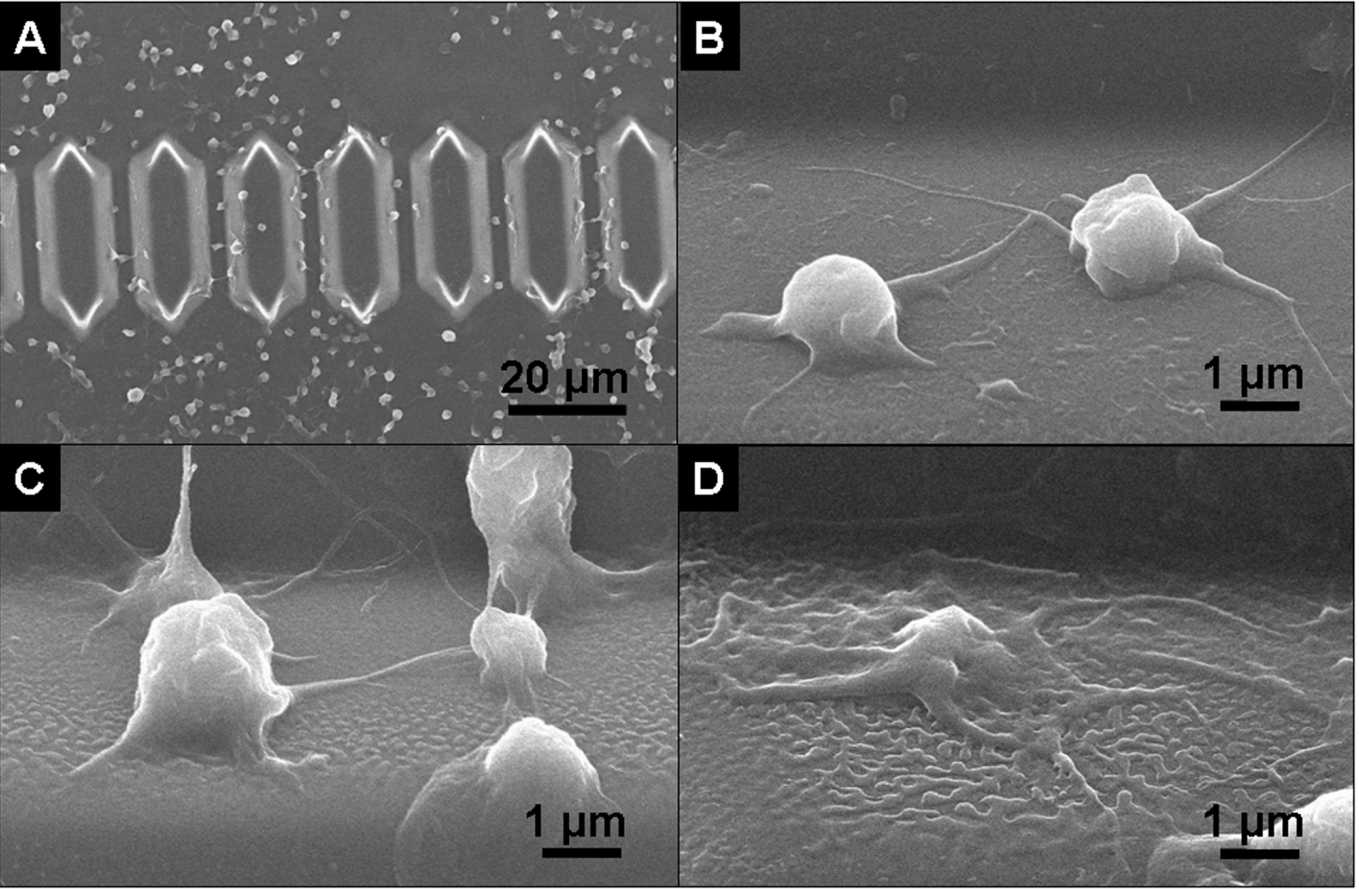
**Platelet morphology**. SEM images of platelets adhered onto topographically structured PMMA surfaces after the BPT measurements. (A) Large view of the micro-channels coated with PMMA3. (B) Detail of a micro-channel coated with PMMA2, (C) PMMA3, (D) PMMA4.

Optical investigation of the chip surface before and after rinsing showed the presence of a higher density of firmly adhered platelets on films with larger feature size. The chip surfaces were investigated using Scanning Electron Microscopy (SEM) after the micro-fluidic experiments and platelet fixation. Figure 4A shows a low magnification image of a part of the chip, where several platelets are seen to adhere along the micro-channel walls and in the areas around them. Closer views of the channel walls are shown in Figures 4B, C and 4D, for the PMMA2, PMMA3 and PMMA4 surfaces respectively. Different platelet morphologies are observed in the three cases. Platelets clearly anchor to the three polymer films. Platelets appeared rounded on PMMA2 and 3, and more flattened and interconnected on the PMMA4 surface. Platelets adhered on PMMA2 film have a smoother surface with respect to those on PMMA3.

### Experiments performed with washed platelets

**Figure 5:**
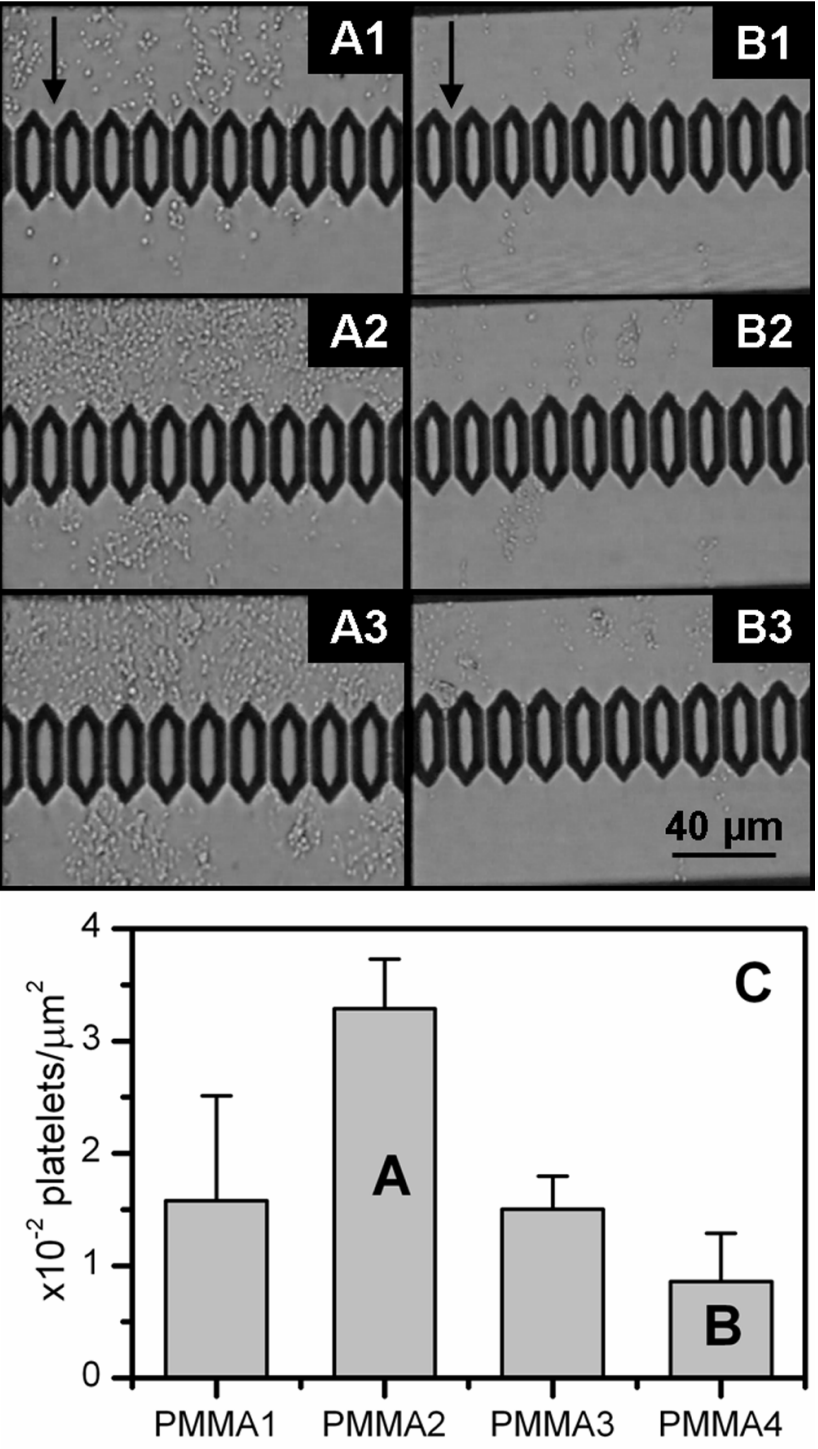
**Microfluidic experiments with washed platelets**. Optical images of (A) PMMA2 and (B) PMMA4 coated chips during the flow experiments performed with washed platelets (WP). The images were taken when (1) 20 μL and (2) 80 μL of WP solution had passed through the channels and (3) after chip rinsing. The arrows indicate the flow direction. (C) Statistical distribution of adhered platelets onto the chips having different surface topographies according to Table 1 after rinsing. Each bar represents the average number of platelets counted over 20 SEM images having the same surface area.

100 μL of washed platelet solution was flowed through the chip channels exhibiting different topographies. The number of platelets that adhered onto each surface appeared to vary depending on the surface feature size. Figures 5A and 5B show optical images of a portion of the chip surface of PMMA2 and PMMA4 respectively during the blood flow and after the chip was rinsed with NaCl solution. The dots visible on the chip surfaces are the platelets. For the duration of the flow, the density of platelet adherence on PMMA4 is lower than on PMMA2. Chip rinsing does not cause a significant detachment of the platelets from the surface, indicating that they are firmly adhered onto the polymer film. Quantitative investigation of platelet density was performed by SEM. Figure 5C shows the statistical distribution of platelet adhered onto the different topographies. Each histogram bar represents the average number of adhered platelets calculated from 20 SEM images with the same surface area (2.99·10^3 ^μm^2^), while the error bars are the standard deviations of each distribution. The average number of adhered platelets per unit area decreases with increasing feature size. However, close examination of the platelets by SEM did not reveal any morphological difference between them in relation to the different topographies.

### Plasma Protein Adsorption Analysis

**Figure 6:**
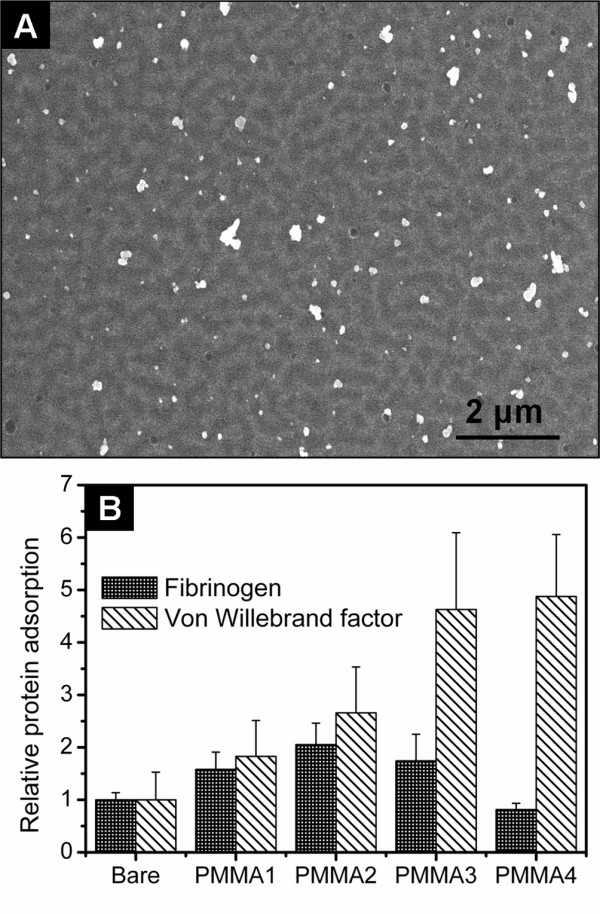
**Plasma protein adsorption analysis**. (A) SEM image of gold and silver labelled von Willebrand factors proteins on a PMMA3 surface. (B) Statistical protein distribution on chips having different surface topographies as reported in Table 1. Each bar represents the average silver surface coverage evaluated over 20 SEM images having the same surface area. The data are normalized to the bare chip surface.

Figure 6A shows a typical SEM image of gold and silver labelled von Willebrand factor adsorbed from platelet poor plasma onto a PMMA structured surface. Figure 6B shows the analysis of fibrinogen and von Willebrand factor distribution on SiO_2 _(reference material), PMMA1, PMMA2, PMMA3 and PMMA4 surfaces. Each histogram bar represents the average of the protein coverage distribution calculated from 20 SEM images having the same area as Figure 6A, while the error bar is the standard deviation of the distribution. The results are normalized to the protein adsorption onto the SiO_2 _surface of a bare chip. Fibrinogen adsorption onto surfaces PMMA1, PMMA2 and PMMA3 is comparable, while it is significantly reduced on PMMA4. Von Willebrand factor adsorption is favoured on structured PMMA surfaces with respect to flat surfaces, and increased on surfaces with larger feature sizes.

## Discussion

We describe a micro-fluidic set-up to study human whole blood interaction with nano-structured polymer films and characterize it in terms of whole blood flow rate, platelet adhesion and protein adsorption on the materials. Compared with conventional techniques, this set-up presents the notable features of requiring a reduced amount of blood, 100 μL, for testing each material. This offers the possibility of using this device in conjunction with human blood. This will eliminate problems associated with using animal blood, such as differences in reactions between different species. Using flow conditions we obtain an impression of the interaction of blood with materials consistent with physiological phenomena; for example, it has been demonstrated that translocating platelets undergo a series of morphological changes in response to increasing fluid shear stress [25], while the activation of plasma proteins such as von Willebrand factors depends on the shear stress they experience [26-28].

One intrinsic limiting factor to note when comparing cell response to micro- and nanostructures is that different techniques are used to create structures on different scales. The use of these different techniques means that micro- and nanostructures can have different surface chemistries. Using polymer demixing only it was possible to produce a set of surfaces having feature sizes varying from the nanometer to the sub-micrometer length scales. XPS chemical analysis of the sample surfaces showed that PMMA is the major (but not sole) component of the film at the interface with blood. The presence of PS domains embedded in the structured films is due to rapid quenching of the solvent during the film spin-coating not allowing the PS and PMMA phases to separate completely. The XPS measurements will encompass the outer surface of the film, to a depth of approximately 10 nm. However, even here a gradient of PS concentration should occur, as the cyclohexane treatment will leach PS from the last few nm of the surface. We are therefore confident that chemical variation between the sample surfaces will be reduced and the major differences in responses to the films can be attributed to surface topography.

Blood flow rate measurements and SEM investigation of platelet morphology concur and indicate that blood interacts differently with the polymer films depending on topography. Data indicates that surfaces with smaller features are potentially less thrombogenic. We can identify three main factors that can influence blood interaction with structured surfaces: 1) different topographies may alter the flow dynamic; 2) different topographies may influence the platelet anchoring and adhesion behaviour onto the polymer surfaces; 3) different topographies may cause dissimilar protein adsorption behaviours.

Surface roughness is found to influence flow dynamics through micro-channels [29,30]; however, numerical simulations indicate that the effects produced are negligible for the geometry of our set up, characterized by Reynolds number < 1 and height of the surface structures relative to the channel height < 0.02.

Platelet anchoring and adhesion behaviour onto the polymer surfaces were studied using both whole blood and washed platelets, i.e. in the absence of blood cells and plasma proteins. Using washed platelets allows the level of activation of the platelets induced by sample handling to be assessed. It is possible that this was sufficient to initiate their adhesion onto the surfaces in absence of the plasma proteins. In the case of experiments performed with whole blood the platelets were seen to adhere and spread preferentially on the polymer films with larger surface feature size.

Conversely, washed platelet adhesion was reduced onto surfaces having larger feature sizes and height of the structures. Optical investigation of the chip surface during micro-fluidic experiments excluded the possibility that lower platelet density on the chip surfaces could be due to the formation of large platelet clusters loosely adhered onto the surface and thus easily removed during the rinsing procedure. We conclude that isolated platelets adhered preferentially on surfaces with smaller feature size. This result, together with the fact that no morphological differences were observed between the washed platelets adhered onto the different topographies, suggests a significant role of the plasma proteins, as well as the blood cells, in the platelet activation mechanism. During experiments performed with whole blood, the erythrocytes and leucocytes may apply a mechanical force to the platelets close to the channel walls when passing through, encouraging their adhesion and activation.

A key protein for regulation dynamic platelet responses is von Willebrand factor. Under shear stress, platelets roll through blood vessels and across surfaces. Platelet rolling is slowed by the formation and breaking of successive translocating bonds with von Willebrand factors. Eventually, if the platelet has slowed enough, stronger bonds form and the platelet firmly anchors to the surface [31,32]. Therefore, surfaces exhibiting a high level of adsorbed von Willebrand factor are more likely to favour platelet adhesion and consequent thrombus formation. Interestingly, lower flow rates were measured for the surfaces exhibiting the higher level of von Willebrand factor absorption. This is particularly interesting, as smaller feature sizes will give a larger surface area, and might intuitively be thought to result in more protein binding. Fibrinogen is a rod-like protein with an important role in thrombogenesis. Our results indicate that fibrinogen adsorption is favoured on surfaces having typical feature size of ~100 nm, while those having larger feature sizes exhibit lower levels of adsorbed fibrinogen.

The two protein we analyzed exhibited opposing adsorption trends with respect to the total surface area of the chips, eliminating this as the sole effect on protein adsorption behaviour on structured surfaces. The reasons why protein adsorption behaviour varies between feature size are not completely understood. The Vroman effect [33] might have significantly contributed to protein displacement and further investigation is required. It has been shown that the supermolecular organization of proteins can be controlled both by surface chemistry and by surface nano-topography [34]. Roach et al[35] showed that proteins of different shapes can associate with a surface in quite dissimilar ways and that surface curvature has an effect on the protein-surface binding affinity and packing density. Their findings suggest that the surface nano-topography may influence both conformation and intermolecular organization of the adsorbed proteins. These effects would be enhanced in the case of rod-like proteins such as the von Willebrand factor and the fibrinogen, both having dimension comparable with the size of the surface features (the von Willebrand factor is estimated about 120 nm in length, while fibrinogen was measured to be about 47 nm × 4 nm [36]). Further investigation is however necessary for elucidating how proteins interact with surface nano-topography under flow condition.

## Conclusion

In conclusion, we have presented a set up for the study of blood interaction with topographically structured polymer surfaces under flow conditions. We demonstrated the utility of this device for measuring the blood flow rate through micro-channels coated with nano-structured PMMA films and we related these values to platelet adhesion and protein adsorption analysis performed on the same surfaces. The results of our investigation indicate that platelet adhesion and consequent thrombus formation is increased onto nano-structured polymer films presenting typical feature sizes of ~400 nm. Interestingly these are the surfaces that present a higher level of von Willebrand factor adsorption. Platelets adhered on such films were found to be flattened and interconnected. No difference in platelet morphology on the various topographies could be observed when platelets were isolated from plasma proteins and blood cells. The fact that adhesion behaviour of washed platelets differed from those in whole blood suggests the significant roles of blood cells and plasma protein adsorption in the activation of adhered platelet in our system. Plasma protein adsorption on nano-structured polymer surfaces was also studied under flow conditions and different adsorption behaviours were found for fibrinogen and von Willebrand factor. We speculate that both the size and the shape of the proteins may have a major role in determining the way these proteins interact with the structured materials.

## Methods

### Nano-structured surfaces preparation

Micro-channel array chips made of silicon (model Bloody6–7, Hitachi Haramachi Electronics Co. Ltd., Japan) with a 20 nm thick silicon oxide layer at the interface with air were used. The 8736 micro-channels of each chip were 30 μm-long and presented a wedge-shaped cross-section, 4.5 μm-deep and between 5 μm and 10 μm-wide. The micro-fluidic chips were coated by spin coating with a polymer film structured by polymer demixing [37,38]. PS (M_w _= 108700, n = 1.06, Polymer Source, Canada) and PMMA (M_w _= 190000, n = 1.8, Polymer Source, Canada) were diluted in toluene at 3.0%, 1.0% and 0.1% (w/v) concentration and stored at room temperature overnight. Polymer blends were made by mixing equal volume portions of stock solutions of each polymer. Polymer films were made by spin coating polymer blends onto the silicon chips at 6000 rpm for 60 s each. The chips were then incubated in cyclohexane for 10 minutes and sonicated for 1 minute to remove the PS molecules from the film surface. The chips were stored in MilliQ H_2_O overnight and sonicated in a 0.9% NaCl solution in MilliQ H_2_O for 1 minute before each micro-fluidic experiment. Samples prepared with the same protocol were dried under a nitrogen flux and characterized by AFM (SPI4000 E-Sweep, Seiko Instruments Inc., Japan) and XPS (Quantum 2000, Physical Electronics, MN, USA). XPS measurements were also performed on a pure PS film for reference. The XPS data were analyzed with the software MultiPak V6.1A (Physical Electronics, Inc.). XPS data analysis and elaboration allowed the estimate that the information contained in each spectrum relate to the outermost 10 nm-thick layer of the polymer film.

### Blood collection

Blood was collected from a healthy individual after informed consent. Heparin was added to a final concentration of 5 IU/mL for the experiments performed with whole blood, which were executed within 30 minutes after blood collection. 1 mL of the collected blood was mixed with 1% (w/v) ethylenediaminetetracetic acid disodium salt (EDTA, Dojindo Laboratories, Japan) in MilliQ H_2_O and analyzed with a particle counter (PCE-170, Erma Inc., Japan) for blood cell counts. The average number of platelets in the whole blood measured was (2.6 ± 0.3)·10^5^/μL.

Washed platelet solution and platelet poor plasma were prepared by centrifuging a 9 : 1 solution of whole blood and 1% EDTA in MilliQ H_2_O in two stages. First the solution was centrifuged at 180 g and the platelet rich plasma was collected. This was then centrifuged at 600 g to separate the platelets from the platelet poor plasma. The collected platelets were gently redispersed in HEPES solution – 140 mM NaCl, 5 mM KCl, 5 mM D-glucose (Wako Pure Chemical Industries, Japan) and 10 nM hidroxyethylpiperazine-ethanosulfonic acid (Research Organics, OH, USA) in MilliQ H_2_O – to a final concentration of 8.9·10^4^/μL. CaCl_2 _(final conc. 0.1 mM) was added just before the measurements.

### Micro-fluidic measurements with whole blood

The micro-fluidic chip was set upside down into the flow chamber of the MC-FAN (KH-3; Hitachi Haramachi Electronics Co. Ltd., Japan) in order to form an array of micro-channels at the boundary with the chamber glass window. For each sample, 100 μL of blood was poured in the central hole of the flow chamber. A tube filled with 0.9% NaCl solution connected the central hole to a volume flow sensor, which was connected to a stopwatch. Another tube connected the flow chamber to a waste bottle, placed 20 cm below the flow volume sensor. The waste bottle was also connected to a pump. When the valve of the pump was opened to air, the blood flowed through the micro-channel array under a pressure difference of 20 cm H_2_O (2.0 kPa) and the blood flow rate was measured by the stopwatch. Simultaneously, the channels were observed by an optical microscope equipped with a CCD camera (LCL-211H, Watec Co. Ltd., Japan) and recorded on a PC. The flow chamber was held by an XY-stage equipped with micrometric screws to observe different chip areas with the camera. After the blood flow, the chip surface was visually investigated and typically some platelets were seen to be adhered to the material's surface. The micro-fluidic chamber was also connected to a bottle of 0.9% NaCl solution used to wash the micro-channels under a 53 kPa pressure to qualitatively evaluate the strength of this adhesion.

The pass-through time of a 0.9% NaCl solution in MilliQ H_2_O was measured before every blood test to evaluate the channel volume variation due to the chip fabrication process and the thickness of the coated polymer film. The presented data were corrected for this effect. Blood coagulation was monitored measuring the blood flow rate on a reference material (bare silicon) before every measurement on a coated chip in order to select consistent results for the data analysis.

### Micro-fluidic measurements with washed platelet

The vertical distance between the MC-FAN volume flow sensor and the waste bottle was set to 10 cm (1.0 kPa), in order to keep shear conditions similar to those experiments performed with the whole blood (We assume blood viscosity 4.4 mPas and washed platelet viscosity similar to that of plasma 1.9 mPas. In separate experiments with the MC-FAN we verified the linearity of the relationship between the flow rate, pressure and inverse of the viscosity). The same measurement protocol described for whole blood was adopted for the micro-fluidic experiment with WP.

### Platelet fixation

After the micro-fluidic measurements, the chips were removed from the flow chamber and washed three times in 1% Dulbecco's Phosphate Buffered Saline without calcium and magnesium (PBS, Nissui Pharmaceutical Co. Ltd., Japan) shaking to remove the loosely adhered cells and platelets. The chips were then stored at 4°C in a 1% gluteraldehyde (Electron Microscopy Science, PA, USA) in H_2_O solution for four hours, rinsed with MilliQ H_2_O, dehydrated via successive incubations in H_2_O/ethanol mixtures with increasing ethanol content and stored at 4°C. The samples were gold coated before investigation by Scanning Electron Microscopy (SEM, S-4800, Hitachi, Japan).

### Plasma protein adsorption analysis

For experiments with platelet poor plasma the vertical distance between the MC-FAN volume flow sensor and the waste bottle was set to 10 cm. The platelet poor plasma was allowed to flow through the chip channels for 1 min. The chips were then rinsed in PBS and stored overnight in a blocking solution – 5% (v/v) Milk Diluent/Blocking Solution Concentrate (KPL, MD, USA) with 2 mM EDTA in MilliQ H_2_O which is filtered by a 0.2 μm membrane filter – at 4°C. The next day, each chip was cut into two parts, and fibrinogen and von Willebrand factor from platelet poor plasma were labelled with gold nanoparticles for SEM analysis; the size of the gold labels and thus their visibility in SEM was enhanced by silver treatment as described in the next section. The chips were finally rinsed with MilliQ H_2_O and dehydrated via successive incubations in H_2_O/ethanol mixtures with increasing ethanol content. The PMMA films exhibited several metal aggregations at their surfaces under SEM investigation. For each micro-fluidic chip we took 20 SEM micrographs from different areas and we counted the number of proteins present at the surface, associating each aggregation with one single adsorbed protein.

### Von Willebrand factor and fibrinogen gold labeling

The labelling protocol comprises three main parts: after rinsing in PBS solution, the chip pieces are incubated in 2 μg/mL of primary antibodies for each protein in 1% (w/v) Ovalbumine solution (INC Biomedicals Inc., OH, USA) for 60 minutes at 37°C. The chips are then stored in a 0.1% gluteraldehyde in H_2_O solution for 30 minutes at 4°C. The chips are incubated for 45 minutes at 37°C in a 10 nm gold labelled secondary antibody solution prepared as suggested by the manufacturer (BBI international, Cardiff, UK). Fibrinogens were labelled with anti-human fibrinogen IgG developed in goat (Sigma, MO, USA) as primary antibody and gold labelled Rabbit anti-Goat IgG as secondary antibody. Von Willebrand factors were labelled using anti-human von Willebrand factor IgG developed in rabbit (Sigma, MO, USA) as primary antibody, and 10 nm gold labelled Goat anti-Rabbit IgG as secondary antibody. The chips are rinsed in MilliQ H_2_O and stored overnight in 1% gluteraldehyde in H_2_O solution. The next day, a silver enhancer procedure (Silver Enhancer Kit, Sigma, MO, USA) was applied for 7 minutes to increase the size of the gold labels and thus their visibility in SEM observation. The final particle size was below 100 nm.

## Competing interests

The author(s) declare that they have no competing interests.

## Authors' contributions

CM conceived and carried out the experiments, analyzed the data and drafted the manuscript. AK participated in the micro-fluidic experiments. NT performed the XPS analysis of the polymer films. MB contributed to the interpretation of the data and the manuscript drafting. AY was essential to the conceiving of the experiments, the interpretation of the results and participated to the drafting of the manuscript. All authors read and approved the final manuscript.
